# Clinical efficacy of adalimumab in Crohn’s disease: a real practice observational study in Japan

**DOI:** 10.1186/s12876-016-0501-9

**Published:** 2016-07-29

**Authors:** Fuminao Takeshima, Daisuke Yoshikawa, Syuntaro Higashi, Tomohito Morisaki, Hidetoshi Oda, Maho Ikeda, Haruhisa Machida, Kayoko Matsushima, Hitomi Minami, Yuko Akazawa, Naoyuki Yamaguchi, Ken Ohnita, Hajime Isomoto, Masato Ueno, Kazuhiko Nakao

**Affiliations:** 1 Department of Gastroenterology and Hepatology, Graduate School of Biomedical Science, Nagasaki University Hospital, 1-7-1 Sakamoto, Nagasaki City, Nagasaki 852-8501 Japan; 2 Department of Gastroenterology and Hepatology, Sasebo City General Hospital, 9-3 Hirase-cho, Sasebo City, Nagasaki 857-8511 Japan; 3 Department of Gastroenterology, National Hospital Organization Nagasaki Medical Center, 2-1001-1 Kubara, Ohmura City, Nagasaki 856-8562 Japan; 4 Department of Gastroenterology and Hepatology, National Hospital Organization Ureshino Medical Center, 2436 Ureshino-cho, Ureshino City, Saga 843-0393 Japan; 5 Department of Gastroenterology and Hepatology, Sasebo Chuo Hospital, 15 Yamato-cho, Sasebo City, Nagasaki 857-1195 Japan; 6 Department of Internal Medicine, Kouseikai Hospital, 1-3-12 Hayama, Nagasaki City, Nagasaki 852-8053 Japan; 7 Department of Internal Medicine, Shunkaikai Inoue Hospital, 6-12 Takara-machi, Nagasaki City, Nagasaki 850-0045 Japan; 8 Integrated Marketing Department, Eisai Co., Ltd., 13-1 Nishigoken-cho, Shinjuku-ku, Tokyo 162-0812 Japan

**Keywords:** Adalimumab, Crohn’s disease, Preventing postoperative recurrence, Japanese patients, Real practice

## Abstract

**Background:**

There are few reports of the efficacy of adalimumab (ADA) for clinical remission and preventing postoperative recurrence in Crohn's disease (CD) in Asian real practice settings. We conducted a Japanese multicenter retrospective observational study.

**Methods:**

We evaluated patients with CD who were treated with ADA at 11 medical institutions in Japan to investigate the clinical efficacy of remission up to 52 weeks and the associated factors to achieve remission with a CD Activity Index (CDAI) < 150. The effects of preventing postoperative recurrence were also evaluated.

**Results:**

In 62 patients, the remission rates were 33.9, 74.2, 75.8, 77.4, and 66.1 % at 0, 4, 12, 26, and 52 weeks, respectively. Although 10 patients discontinued treatment due to primary nonresponse, secondary nonresponse, or adverse events, the ongoing treatment rate at 52 weeks was 83.9 %. Comparison of remission and non-remission on univariate analysis identified colonic type and baseline CDAI value as significant associated factors (*P* < 0.05). In 16 patients who received ADA to prevent postoperative recurrence, the clinical remission maintenance rate was 93.8 % and the mucosal healing rate was 64.3 % during a mean postoperative follow-up period of 32.3 months.

**Conclusions:**

ADA effectively induced remission and prevented postoperative recurrence in patients with CD in a real practice setting.

## Background

Crohn's disease (CD), an inflammatory bowel disease (IBD), is an intractable disease of unknown etiology [1]. CD is progressive and markedly impairs patient quality of life due to its associated symptoms such as diarrhea, abdominal pain, fever, and surgery [2–4]. Although there is currently no cure for CD, the inflammatory cytokine tumor necrosis factor-α (TNF-α) is involved in clinical condition [5], against which anti-TNF-α monoclonal antibody (anti-TNF-α antibody) is highly effective [6, 7]. For the treatment of CD, infliximab (IFX), a chimeric antibody and infusion drug, first appeared as an anti-TNF-α antibody, followed by adalimumab (ADA), a fully humanized antibody and subcutaneous drug. IFX and ADA were highly effective in large-scale studies and have already been widely used worldwide [8–15].

Although the clinical features of CD in Asia is relatively similar to that of North America or Europe, there are some differences such as a higher prevalence of males and ileo-colonic type, less familial clustering, extra-intestinal manifestations, and surgical rates. As for genetics, nucleotide oligomerization domain-2 (NOD2) variants and autophagy-related 16-like 1(ATG16L1) variants, which have been firmly associated with CD in the West have not been detected in the patients with CD in Asia [16]. Taking account of these differences, the data from Asia is important in spite of the numerous data from West. However, ADA received approval later in Asian countries including Japan than in the Europe and US; therefore, there are limited reports on its efficacy in Asian real practice settings [17–20]. Watanabe et al. reported that clinical remission rate at week 4 in the induction therapy was 33.3 %. Seventy percentage of patients achieved decrease in CDAI ≥ 70 points and the rest was unresponsive at week 4 in the induction therapy [17]. Furthermore, intestinal resection is commonly required in patients with CD and often leads to repeated surgery; thus, it is important to prevent postoperative recurrence [21, 22]. Recent reports indicated that IFX and ADA effectively prevent postoperative recurrence [23–26]. However, no report to date has demonstrated the ability of ADA to prevent postoperative recurrence in Asia. Under these circumstances, we conducted a multicenter observational study in Japan to evaluate ADA efficacy and safety as well as its ability to prevent postoperative recurrence in patients with CD in real practice settings.

## Methods

### Study design

The Nagasaki observational study of adalimumab is a multicenter retrospective observational study of patients with IBD receiving ADA treatment at a total of 11 medical sites including Nagasaki University Hospital and its related facilities. This study was reviewed and approved by the Nagasaki University Hospital Ethics Committee before its initiation.

### Patients

The study included all patients who received ADA for the treatment of CD at a total of 11 study sites including Nagasaki University Hospital and its related facilities between November 2010 and January 2014. Patients who failed to complete 52 weeks of follow-up due to relocating were excluded from the analysis.

### Treatment protocol

ADA was administered at an initial dose of 160 mg and a second dose of 80 mg with a 2-week induction interval. Thereafter, ADA 40 mg was administered every other week as maintenance therapy. ADA dose intensification was not included in this study because it is not currently approved in Japan.

### Data collection

A shared common database was used to collect demographic and clinical data. Data collected at baseline were sex, age, disease duration, disease extension, history of operation, smoking habits, concomitant fistula or anal lesion, previous infliximab therapy, concomitant medications or elemental diet at baseline, C-reactive protein (CRP) levels, and the Crohn’s disease activity index (CDAI) [27]. Clinical activity was evaluated using the CDAI after 4, 12, 26, and 52 weeks of treatment. The date of and reason for ADA discontinuation, requirement of further rescue therapy, and adverse events were also recorded.

### Definition

We defined remission as a CDAI < 150. Non-remission was defined as discontinuation due to a lack of efficacy, adverse event, or other reason. In addition, we evaluated the ability of ADA to prevent postoperative recurrence in patients with any recurrence risk factor [28] such as smoking, penetrating disease, history of prior resection, and short disease duration (<10 years) after intestinal resection. Clinical remission was defined as a CDAI < 150, and mucosal healing was confirmed by a Rutgeerts score of i0 or i1 [29].

### Endpoints

The primary endpoints of the study were rates of clinical remission at 4, 12, 26, and 52 weeks in the intention-to-treat (ITT) population, and the proportion of patients still receiving ADA therapy at the end of the first year. The secondary endpoints included the maintenance rate of clinical remission and mucosal healing in the patients who received ADA treatment for the prevention of postoperative recurrence.

### Statistical analysis

All efficacy analyses were performed on an ITT basis. Statistical analysis was performed with a Chi-square test or Fisher's exact test for categorical data and Student’s *t*-test or the Mann-Whitney *U*-test for continuous variables. Factors identified as having significant differences on univariate analysis were further assessed by multivariate analysis with logistic regression. *P* values < 0.05 were considered statistically significant.

## Results

### Patient demographics

**Fig. 1:**
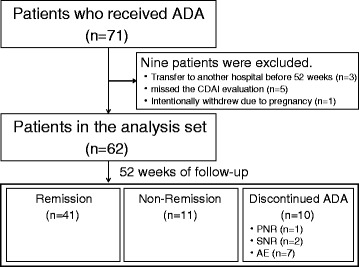
Flowchart of patient disposition. ADA; adalimumab, CDAI; Crohn’s Disease Activity Index, PNR; primary nonresponse, SNR; secondary nonresponse, AE; adverse event

Of 71 patients who received ADA treatment during the study period, 62 were included in the analysis set, while the other nine were excluded: three who failed to complete follow-up due to transfer to another hospital before 52 weeks, five who missed the CDAI evaluation, and one who intentionally withdrew from treatment due to pregnancy (Fig. 1). The baseline characteristics of the 62 patients are shown in Table 1. The mean age was 33.1 years, 74.2 % of the patients were men, the median disease duration was 96 months, and 32.3 % of the patients had a disease duration < 2 years. Prior treatment consisted of IFX in 53.2 % of the patients; of them 33.3 % were on a double IFX dose (10 mg/kg). The mean baseline CDAI was 185.1 points, and 33.9 % of the patients had a CDAI < 150 before ADA treatment, including those who required a medication switch due to IFX intolerance.

**Table 1:** Patients’ baseline characteristics

Patient number	62
Age (years)	33.1 ± 10.3
Gender (male)	74.2 % (46/72)
Disease duration (months)	96.0 (0.0–480.0)
Disease duration < 2 years	32.3 % (20/62)
Disease location	
Ileo	14.5 % (9/62)
Ileocolonic	74.2 % (46/62)
Colonic	11.3 % (7/62)
Surgery required	62.9 % (39/63)
Extra fistula	34.4 % (21/61)
Intra fistula	8.2 % (5/61)
Perianal disease	48.4 % (30/62)
Smoking	17.3 % (9/52)
Concomitant use	
5-Aminosalicylates	91.9 % (57/62)
Steroids	9.7 % (6/62)
Immunomodulators	24.2 % (15/62)
Elemental diet	62.9 % (39/62)
IFX experience	
Experience with IFX	53.2 % (33/62)
Double dose	33.3 % (11/33)
Duration of IFX use (months)	19.0 (1.0–100)
Reason for switching	
PNR	6.3 % (2/32)
SNR	46.9 % (15/32)
Intolerance	43.8 % (14/32)
Others	3.1 % (1/32)
Baseline CDAI (points)	185.1 ± 76.4
Baseline CRP (mg/dL)	0.74 (0.00–6.86)

Parametric variables are shown as mean ± standard deviation or median (range)

*IFX* infliximab, *PNR* primary nonresponse, *SNR* secondary nonresponse, *CDAI* Crohn’s Disease Activity Index, *CRP* C-reactive protein

### Clinical efficacy

**Fig. 2:**
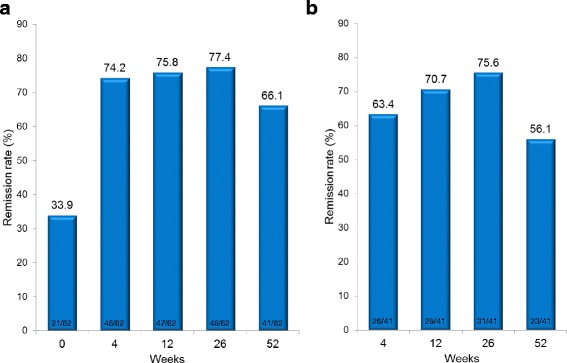
Remission rate after up to 52 weeks of adalimumab treatment. The proportion of patients who achieved remission with a Crohn’s Disease Activity Index (CDAI) < 150 is shown. **a** Remission rate of all 62 patients. **b** Remission rate of all 41 patients with a baseline CDAI ≥ 150. Ten patients who discontinued the study treatment were categorized into the non-remission group

**Fig. 3:**
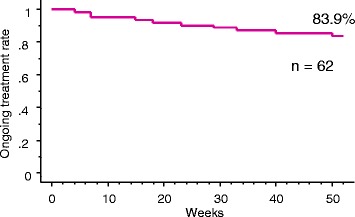
Ongoing adalimumab (ADA) treatment rate. A total of 10 patients discontinued treatment due to primary nonresponse (*n* = 1), secondary nonresponse (*n* = 2), or an adverse event (*n* = 7). The ADA ongoing treatment rate at 52 weeks was 83.9 %

In all 62 patients, the remission rates were 33.9, 74.2, 75.8, 77.4, and 66.1 % at 0, 4, 12, 26, and 52 weeks, respectively (Fig. 2a). In the 41 patients whose baseline CDAI was ≥150, the remission rates were 63.4, 70.7, 75.6, and 56.1 % at 4, 12, 26, and 52 weeks, respectively (Fig. 2b). Of the 62 patients, 10 discontinued treatment due to primary nonresponse (PNR) (*n* = 1), secondary nonresponse (SNR) (*n* = 2), or adverse event (*n* = 7). The ongoing ADA treatment rate at 52 week was 83.9 % (Fig. 3).

### Efficacy predictor

An univariate analysis was performed to compare 41 patients who achieved remission at 52 weeks of ADA treatment (remission group) with 21 patients who failed to achieve remission with a CDAI ≥ 150 or discontinued ADA treatment before 52 weeks (non-remission group) (Table 2). Significant differences between groups were detected only for the colonic type (*P* = 0.0387) and baseline CDAI (*P* = 0.0236). The remission group had a slightly lower median disease duration and included more patients with a disease duration < 2 years, but the difference was not statistically significant. A multivariate analysis was performed for the two factors, colonic type and baseline CDAI, identified significant differences from the univariate analysis, but both were not found to be significant (Table 3).

**Table 2:** Comparison of remission and non-remission groups at 52 weeks

	Remission (*n* = 41)	Non-remission (*n* = 21)	*P* value
Age (years)	32.1 ± 10.5	34.9 ± 10.1	0.3201^a^
Gender (male)	75.6 % (31/41)	71.4 % (15/21)	0.7648^c^
Disease duration (months)	87.0 (0.0–480.0)	144.0 (0.0–336.0)	0.3964^b^
Disease duration < 2 years	36.6 % (15/41)	23.8 % (5/21)	0.3962^c^
Colonic type	4.9 % (2/41)	23.8 (5/21)	*0.0387^c^
Surgery	61.0 % (25/41)	66.7 % (14/21)	0.7837^c^
Extra fistula	30.0 % (12/40)	42.9 % (9/21)	0.3980^c^
Intra fistula	7.5 % (3/40)	9.5 % (2/21)	>0.9999^c^
Perianal disease	46.3 % (19/41)	52.4 % (11/21)	0.7895^c^
Smoking	17.6 % (6/34)	16.7 % (3/18)	>0.9999^c^
Concomitant use			
5-Aminosalicylates	87.8 % (36/41)	100 % (21/21)	0.1569^c^
Steroids	9.8 % (4/41)	9.5 % (2/21)	>0.9999^c^
Immunomodulators	22.0 % (9/41)	28.6 % (6/21)	0.7548^c^
Elemental diet	65.9 % (27/41)	57.1 % (12/21)	0.5829^c^
IFX experience			
Experience with IFX	51.2 % (21/41)	57.1 % (12/21)	0.7895^c^
Double dose	28.6 % (6/21)	41.7 % (5/12)	0.4713^c^
Duration of IFX use (months)	19.5 (1.0–84.0)	10.0 (1.0–100)	0.7818^b^
Reason of switch			0.5700^d^
PNR	10.0 % (2/20)	0.0 % (0/12)	
SNR	45.0 % (9/20)	50.0 % (6/12)	
Intolerance	40.0 % (8/20)	50.0 % (6/12)	
Other	5.0 % (1/20)	0.0 % (0/12)	
CDAI at baseline (points)	169.5 ± 76.8	215.4 ± 67.2	*0.0236^a^
CRP at baseline (mg/dL)	0.70 (0.00–6.86)	0.88 (0.02–6.34)	0.4436^b^

Parametric variables are shown as mean ± standard deviation or median (range)

*IFX* infliximab, *PNR* primary nonresponse, *SNR* secondary nonresponse, *CDAI* Crohn’s Disease Activity Index, *CRP* C-reactive protein

*P < 0.05

^a^Student’s *t*-test; ^b^Mann-Whitney *U*-test; ^c^Fisher’s exact test; ^d^Chi-square test

**Table 3:** Predictors of remission at 52 weeks of adalimumab treatment (multivariate analysis)

Factor	Odds ratio (95 % CI)	*P* value
CDAI at baseline	0.993 (0.985–1.000)	0.0577
Colonic type	0.207 (0.035–1.230)	0.0833

*CI* confidence interval, *CDAI* Crohn’s Disease Activity Index

### Prevention of postoperative recurrence

The baseline characteristics, presence of risk factors, clinical remission after ADA treatment, and mucosal healing by endoscopy of the 16 high-risk patients who received ADA treatment for the prevention of postoperative recurrence after intestinal resection are shown in Table 4. Each patient had an average of 2.1 of four recurrence risk factors including smoking, penetrating disease, previous resection, and disease duration < 10 years. The maintenance rate of clinical remission up to the last observation period (a mean of 32.3 months) was 93.8 % (15/16 patients). Endoscopy was performed in 14 patients at a mean 25.3 months, and mucosal healing was confirmed in 64.3 % (9/14 patients). Clinical remission was confirmed in four of five patients who experienced endoscopic relapse. Of the three patients whose relapse was confirmed with a Rutgeerts score of i3 or i4, two had experience with nonresponse to IFX treatment, while the patient with a Rutgeerts score of i4 had all four risk factors.

**Table 4:** Results of ADA treatment for the prevention of postoperative recurrence

Basic characteristic							Risk factor					Clinical remission (last examination)		Endoscopy (last examination)		
Patient	Gender	Age (years)	Disease duration (months)	Disease location	Previous IFX	Time to ADA after resection (days)	Smoking	Penetrating disease	Previous resection	Disease duration (<10 years)	Numbers of risk factors	Time to last examination (months)	Remission	Time to Endoscopy(months)	Rutgeerts score	Mucosal healing
1	M	33	153	Ileocolonic	○	43	×	○	○	×	2	48	○	20	i0	○
2	M	32	77	Ileocolonic	○	18	○	○	○	○	4	50	○	44	i4	×
3	M	38	96	Ileocolonic	×	23	○	○	×	○	3	49	○	47	i0	○
4	M	30	178	Ileo	○	57	×	○	○	×	2	46	×	37	i3	×
5	M	29	3	Ileocolonic	×	40	○	×	×	○	2	40	○	28	i2	×
6	M	50	276	Ileo	×	49	×	×	○	×	1	32	○	23	i0	○
7	M	19	8	Ileocolonic	×	15	○	×	×	○	2	38	○	22	i0	○
8	M	50	480	Ileocolonic	○	23	×	○	○	×	2	38	○	38	i1	○
9	M	34	180	Ileo	○	60	○	×	○	×	2	31	○	8	i0	○
10	M	33	120	Ileo	×	240	×	○	×	○	2	29	○	28	i2	×
11	M	43	204	Ileocolonic	×	73	×	○	○	×	2	32	○	-	-	-
12	M	47	36	Ileocolonic	×	53	×	○	○	○	3	24	○	-	-	-
13	M	23	18	Ileocolonic	×	22	×	○	×	○	2	15	○	8	i0	○
14	F	45	396	Ileocolonic	×	82	×	○	○	×	2	24	○	24	i3	×
15	M	27	3	Ileocolonic	×	10	×	×	×	○	1	16	○	15	i0	○
16	F	31	18	Ileocolonic	×	38	×	○	×	○	2	12	○	12	i0	○
Total	M/F: 14/2	35.3	140.4	4/12	31.3 % (5/16)	52.9	31.3 % (5/16)	68.8 % (11/16)	56.3 % (9/16)	56.3 % (9/16)	2.1	32.3	93.8 % (15/16)	25.3	-	64.3 % (9/14)

Parametric variables as total are shown as mean or rates

*ADA* adalimumab, *IFX* infliximab

### Safety

In the assessment of up to 52 weeks, adverse events were reported in a total of eight patients: pancytopenia, sepsis, lupus-like reaction, hepatic function disorder, recurrent upper respiratory tract infection, ss-DNA antibody positive, catheter infection, and rash in one patient each. ADA treatment was discontinued due to adverse events in all seven patients except the one with the rash. All events were resolved under observation after ADA discontinuation or with treatment. There was no report of malignant tumor development or death.

## Discussion

ADA has been demonstrated effective against CD in the CLASSIC I [11], CLASSIC II [13], GAIN [12], CHARM [14], and EXTEND [15] placebo-controlled double-blind studies. In Japan, its clinical efficacy was also demonstrated in a placebo-controlled double-blind study [17], and ADA has been available for clinical use since October 2010. However, limited reports to date are available on usage data in real practice settings in Japan and other Asian countries. Ishida, et al. [19] and Miyoshi, et al. [20] reported results from a single center study with limited sample sizes of 28 and 45 subjects, respectively. Therefore, we investigated the results of ADA treatment for CD in real practice settings in this multicenter observational study. The remission rates from 4 to 52 weeks were similar to those reported by Ishida, et al. [19] and Miyoshi, et al [20]. ADA was more effective in real practice settings than in a clinical study with limited patients based on various exclusion criteria and a CDAI of 220–450 [17].

This study identified CD location and baseline CDAI as factors associated with ADA efficacy at 52 weeks. A study by Cohen, et al. [30] also indicated a slightly higher nonresponse rate in patients with the colonic type but with no significant difference. To our knowledge, only the current study demonstrated lower ADA efficacy in patients with the colonic type. We believe that the effects of TNF-α may be greater in patients with lesions in the small intestine, but no significant difference was detected on multivariate analysis, so this issue requires further investigation. Other factors including previous IFX treatment [31, 32] and disease duration [33, 34] are reportedly associated with ADA efficacy, but this study did not show a significant difference in these factors. This may be due to an insufficient sample size and the fact that more patients had fewer disease activity events such as intolerance with previous IFX treatment. The combined effect of immunomodulator and ADA has yet to be confirmed because of conflicting reports [35–38], and no difference was noted in this study either. Thus, prospective comparative studies of ADA like the IFX SONIC study [39, 40] are needed. The combined effect of an elemental diet and IFX has also been reported [41–43], but no difference was observed in this study. Serum ADA concentration and involvement in efficacy of anti-adalimumab antibody (AAA) have been highly reported recently [44–49], but we could not evaluate these parameters in the current study.

We also investigated the ability of ADA to prevent postoperative recurrence. Several reports have been published to date in Europe or the US [25, 50–52], but there are few reports from Asian countries. This study confirmed that ADA effectively prevented postoperative recurrence in a small sample of 16 patients. While a meta-analysis indicated the efficacy of anti-TNF-α antibody for preventing postoperative recurrence [26], the use of anti-TNF-α antibody has a cost-benefit performance issue [53] and is recommended for patients at high risk and those in whom relapse was observed in postoperative monitoring [54]. The POCER study recently reported on the usefulness of postoperative risk factor–based therapeutic stratification, endoscopic monitoring, and therapeutic intensification [55]. However, in the POCER study, thiopurine was used as the first-line treatment even for high-risk patients, while the active care group with endoscopic evaluation and therapeutic intensification had a relatively high recurrence rate. A greater number of risk factors was associated with a higher relapse rate. Our study also indicated more risk factors in patients who showed endoscopic recurrence with a Rutgeerts score of i3 or i4. Therefore, positive use of anti-TNF-α antibody may require consideration for high-risk patients. Recent studies reported the usefulness of fecal calprotectin as a parameter in postoperative monitoring [56, 57]. Postoperative monitoring and therapeutic optimization as considerations of the burden of examination and cost-benefit relationship are required.

This study has several limitations. First, it had an insufficient sample size of unselected patients with heterogeneous baseline characteristics in a real practice setting. Therefore, it was difficult to determine the efficacy-associated factors. Second, the assessment of prevention of postoperative recurrence was made only in a small group of 16 patients, the timing of ADA introduction and endoscopic examination was inconsistent, and endoscopy was not performed for some patients. Therefore, further larger-scale studies with strict evaluation methods are required to validate our results.

## Conclusions

Even with the above-mentioned limitations, we concluded in this study that ADA effectively provided clinical remission and prevented postoperative recurrence of CD in real practice settings.

## Abbreviations

AAA, anti-adalimumab antibody; ADA, adalimumab; anti-TNF-α antibody, anti-TNF-α monoclonal antibody; CD, Crohn's disease; CDAI, CD Activity Index; CRP, C-reactive protein; IBD, inflammatory bowel disease; IFX, infliximab; PNR, primary nonresponse; SNR, secondary nonresponse; TNF-α, tumor necrosis factor-α.
